# Dynamic imaging of the growth plate cartilage reveals multiple contributors to skeletal morphogenesis

**DOI:** 10.1038/ncomms7798

**Published:** 2015-04-13

**Authors:** Yuwei Li, Vikas Trivedi, Thai V. Truong, David S. Koos, Rusty Lansford, Cheng-Ming Chuong, David Warburton, Rex A. Moats, Scott E. Fraser

**Affiliations:** 1 Division of Biology and Biological Engineering, California Institute of Technology, Pasadena, California 91125, USA; 2 Department of Molecular and Computational Biology, University of Southern California, Los Angeles, California, USA; 3 Developmental Biology and Regenerative Medicine Program, Saban Research Institute, Children's Hospital Los Angeles, Los Angeles, California 90027, USA; 4 Department of Radiology, The Saban Research Institute, Children's Hospital Los Angeles, Los Angeles, California 90027, USA; 5 Department of Pathology, Keck School of Medicine, University of Southern California, Los Angeles, California, USA; 6 Department of Biomedical Engineering, Viterbi School of Engineering, University of Southern California, Los Angeles, California, USA

## Abstract

The diverse morphology of vertebrate skeletal system is genetically controlled, yet the means by which cells shape the skeleton remains to be fully illuminated. Here we perform quantitative analyses of cell behaviours in the growth plate cartilage, the template for long bone formation, to gain insights into this process. Using a robust avian embryonic organ culture, we employ time-lapse two-photon laser scanning microscopy to observe proliferative cells' behaviours during cartilage growth, resulting in cellular trajectories with a spreading displacement mainly along the tissue elongation axis. We build a novel software toolkit of quantitative methods to segregate the contributions of various cellular processes to the cellular trajectories. We find that convergent-extension, mitotic cell division, and daughter cell rearrangement do not contribute significantly to the observed growth process; instead, extracellular matrix deposition and cell volume enlargement are the key contributors to embryonic cartilage elongation.

## Abstract (web summary)

It is largely unknown how individual cell behaviours translate into collective cell action that underlies bone morphogenesis. Here the authors perform quantitative imaging of the long bone cartilage growth plate and identify the key cellular mechanisms of cartilage morphogenesis.

## Graphical abstract

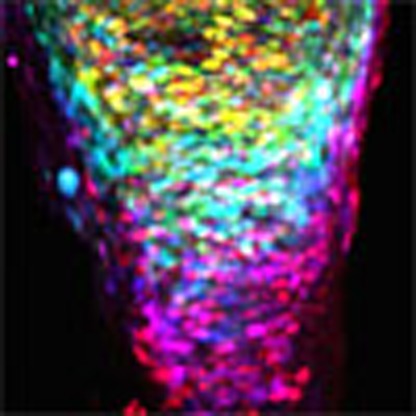

Among the diverse skeletal elements, the growth plate cartilage of long bones (limb skeleton) is ideal for 4D (*xyz* and *t*) analyses of cell behaviours due to its largely unidirectional growth and cylindrical shape. Growth is accompanied by the progressive differentiation of chondrocytes along the proximo-distal axis (PDA; Fig. 1a,b)^1^,^2^,^3^. Residing close to the articular cartilage are the round and dispersed chondrocytes of the resting zone, which are progenitor cells that differentiate into the elliptical and organized chondrocytes of the proliferative zone (PZ)^1^,^2^,^3^. During this transition process, chondrocytes deposit collagen II (*col2*), a specialized extracellular matrix (ECM) protein, into the intercellular space. After a period of cell division, the proliferative chondrocytes differentiate sequentially into the enlarged chondrocytes in the prehypertrophic and hypertrophic zone (HZ). The terminally differentiated HZ chondrocytes deposit another type of ECM protein, collagen X (*col10*), which serves as a scaffold for the invasion of osteoblast cells; the osteoblasts lay down a calcified bony matrix for long bone maturation^1^,^2^,^3^.

Multiple signalling pathways, including Indian hedgehog (*Ihh*)^4^,^5^, parathyroid hormone-related peptide (*Pthrp*)^4^,^5^, bone morphogenetic proteins (Bmp)^6^, fibroblast growth factors (*FGFs*)^7^ and wingless/int-1 molecules (*Wnt*)^8^, form a molecular regulatory circuit controlling cartilage growth and morphogenesis. Significant work in the past, based on static imaging, has suggested the importance of convergent-extension (CE) like behaviours of the PZ cells^9^,^10^, volume increase of the HZ cells^11^,^12^,^13^ and deposition of ECM^12^ to adult cartilage elongation. However, the specific contributions and relationships of the various cellular processes to embryonic cartilage elongation have yet to be fully defined. Static imaging studies face two general challenges for dissecting tissue morphogenesis: first, fixing and processing of tissues can alter cell sizes and the spaces between them in a non-uniform manner; second, dynamic cellular processes can only be inferred from static studies. In the absence of tools for visualizing the various cellular processes and systematically dissecting their roles, it is difficult to understand how individual cell behaviours are translated into the collective cell behaviour that underlies tissue morphogenesis.

To meet these challenges, we perform quantitative imaging on live tissue and generate predictive models to extract information on the underlying cellular dynamics. Quantitative live optical imaging allows the longitudinal visualization of individual cells and the quantitative description of their behaviours in 4D^14^,^15^,^16^. *In silico* modelling takes these quantitative measures of PZ cell features and behaviours (for example, length and speed of cell displacement, absolute and relative orientation of cell division, the rate of ECM deposition and cell volume change) and creates predictions that can then be tested using quantitative imaging tools. Our closed loop analysis reveals that embryonic cartilage elongation is highly coordinated, with critical contributions from two types of cell morphogenesis in the PZ: ECM deposition and cell volume enlargement.

## Results

### Avian metacarpal culture for 4D imaging of cartilage elongation

**Figure 1:**
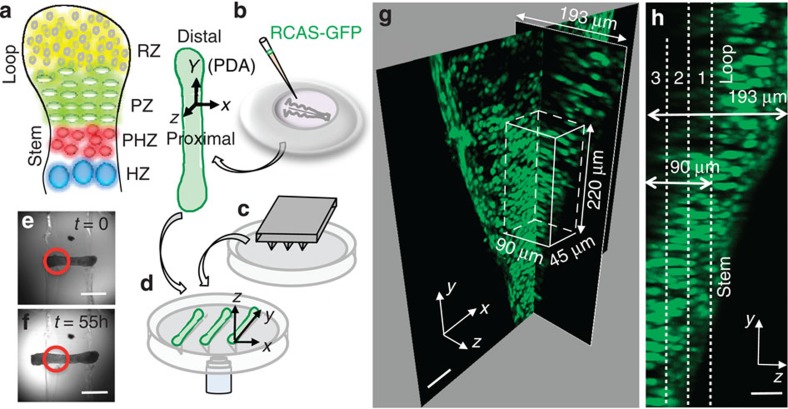
Live imaging of cartilage growth. (**a**) The growth plate cartilage displays a stem-loop shape and contains four zones along the PDA: resting (RZ), proliferative (PZ), prehypertrophic (PHZ) and hypertrophic (HZ) zones. (**b**–**d**) Schematic diagrams of metacarpal culture and imaging. (**b**) Chick forelimb bud was injected with RCAS-GFP and the viral-infected metacarpal was dissected for organ culture. (**c**) A mold (grey) was inserted into molten agarose on an imaging dish to create grooves (**d**), which were used to hold the metacarpals stably for imaging through the objective (light blue) on an inverted laser scanning microscope. (**e**) Top view of the metacarpal sitting in the groove. (**f**) The same metacarpal exhibiting similar extension on both sides after live imaging for 55 h. The red circles in (**e**) and (**f**) depict the region imaged. (**g**) Two orthogonal sections shown in the 3D rendered image of the GFP-expressing (green) metacarpal at *t*=29 h (chosen for the best depiction) of live imaging. The white cuboid drawn shows the approximate region selected for the PZ cell tracking. (**h**) Side view of the same sample. The middle 90-μm region spanning both stem and loop parts was digitally sliced into three sections along the imaging axis (Methods) to facilitate cell segmentation. Scale bars, (**e**,**f**) 500 μm, (**g**) 50 μm and (**h**) 20 μm.

To permit our quantitative imaging analyses of skeleton shaping and the underlying cellular processes in the PZ, we established an organ culture system that supported normal growth and permitted longitudinal imaging of the live specimen. The metacarpal of the forelimb provides an excellent experimental system, as the embryonic day 8 (E8) chick metacarpal is largely PZ and is sufficiently thin that nutrients can penetrate to the chondrocytes (Supplementary Fig. 1a–c)^17^, resulting in normal growth when isolated in culture (Supplementary Fig. 1d,e). We injected replication-competent avian retrovirus into the donor forelimb bud at E3 (Fig. 1b), so that the chondrocytes in the metacarpal harvested at E8 are globally labelled with green fluorescent protein in cytoplasm (cytoplasmic-GFP) (Supplementary Fig. 2).

To stabilize the metacarpal for long-term imaging, it was mounted in grooves cast in agarose, using a custom-designed plastic mold based on the metacarpal dimensions (Fig. 1c,d). The agarose provides a non-stick surface that permits natural tissue elongation and morphogenesis. To avoid the possibility that the enlarged ends of the metacarpal (Fig. 1a) might lodge in the agarose, we removed the agarose surrounding the ends; thus, only the more cylindrical stem region was in contact with the agarose groove (Fig. 1e).

### 4D imaging and segmentation of the PZ cells

To non-invasively visualize cells in multiple layers of the explanted bone, we used two-photon laser scanning microscopy (TPLSM)^18^, which can image cells deeper in the tissue than conventional confocal laser scanning microscopy. Image stacks were collected of one end of the cultured metatarsal hourly for 55 h, to a depth that reached half the thickness (Supplementary Movie 1). The illuminated and unexposed half of the metacarpal exhibited similar length extension (Fig. 1f), suggesting there is little if any photo-toxicity or detrimental effects of being cultured on the microscope stage.

**Figure 2:**
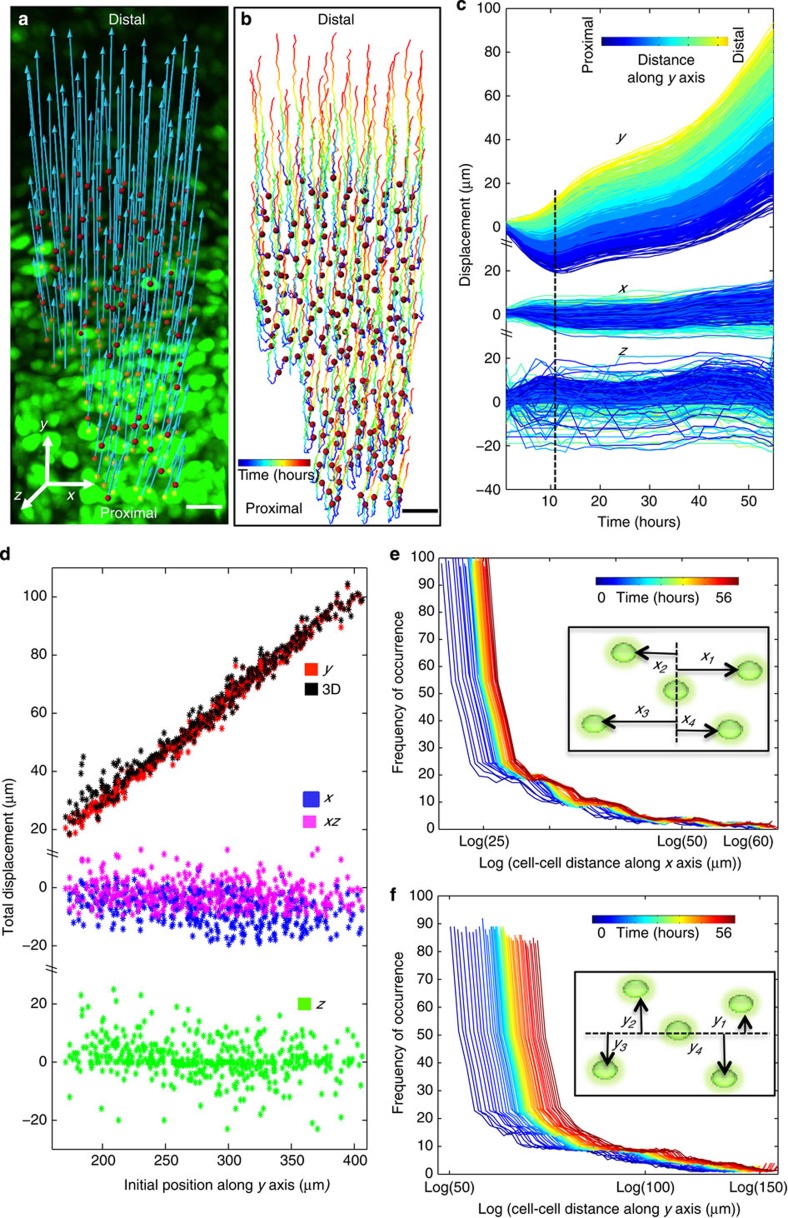
Cells undergo collective spreading displacement during cartilage growth. (**a**,**b**) Cells in the proliferative zone (PZ) were segmented (red dots), and their net displacement vectors (blue lines in **a**) and trajectories (colored lines in **b**) were mapped, showing strong orientation of the cell displacement towards the distal end of the tissue. (**c**,**d**) Analysis of individual cell trajectories. (**c**) Cell displacement over time relative to its initial *y* position (colour-coded as shown in the top inset), showing the largest displacement along the *y* axis and smaller along the *x* and *z* axes. (**d**) Total cell displacement (*t*=55 h) along different axes and planes relative to their initial *y* positions. Cell displacements along the *y* axis account for most of the displacements in 3D with linear increase in magnitude according to their initial *y* positions (*R*^2^=0.986), as expected if the motion of the cells depends both on local changes and similar changes happening more proximally. (**e**,**f**) Analysis of cell-cell distance change. The mean of centre-to-centre distance between all possible pairs of cells at any given time was measured (as indicated in the insets), and the distributions of those means for all cells over time (colour-coded) were plotted along the *x* (**e**) and the *y* axes (**f**) on a semi-log scale (to amplify the increase in mean over time graphically). More increase in cell-cell distance along the *y* axis as compared with the *x* axis implies an anisotropic spreading behaviour of the PZ cells; *n*=472 cells in (**c**–**f**). Scale bars (**a**,**b**), 50 μm.

We identified the PZ cells based on their positions in the live tissue (Supplementary Fig. 1f). To quantitatively define these cell behaviours, we performed 3D spot segmentation of individual cells, so that they could be tracked over time in a 220 × 90 × 90 μm^3^ region (Fig. 1g,h; Fig. 2a,b; Supplementary Movies 2–4).

We estimated that our segmentation and tracking is able to accurately track 95% of the cells in the volume. This was validated by labelling the post-imaging sample with phalloidin and finding that 97% of the cells identified in this way were GFP labelled (Supplementary Fig. 2). In addition, all computed trajectories were manually checked and corrected for possible errors. Our processing was able to identify 481 cells, out of which we eventually analysed 472 cells (98%). Thus, we conclude that our segmentation and tracking is able to accurately cover 95% (98 of 97% labelled cells) of all the cells.

### Anisotropic spreading of the PZ cells

To analyze and display the 4D trajectories, we defined a Cartesian coordinate system aligned with the shape of the metacarpal (Figs 1b and 2a): The PDA was defined as the *y* axis, with the middle of the tissue taken as the origin; the axis perpendicular to the long axis in the imaging plane was defined as the *x* axis; the axis coming out (along the light path) and orthogonal to the image plane was defined as the *z* axis.

Decomposition of cellular trajectories using this Cartesian coordinate system revealed a clear bias to the spreading behaviour, with the most significant movement along the *y* axis (Fig. 2c). This *y* axis motion occurred in two phases, with many cellular trajectories moving proximally in the first 10 h of imaging; thereafter, all trajectories coursed distally. Colour-coding the trajectories according to the initial positions of the cells revealed an inherent order, with the displacements increasing progressively from the proximal to the distal end in the second phase (Fig. 2c). Such a coordinated pattern was not observed in the *x* and *z* directions (Supplementary Fig. 3a,b), suggesting that the cell displacements are mainly determined by their initial positions along the PDA.

### Additive nature of the anisotropic cell spreading

The orderly spreading of cells suggests some cooperation between cells, the nature of which needed further investigation. By analysing the total *y* displacement of each cell with respect to its initial *y* position, we found a linear relationship (*R*^2^=0.986) (Fig. 2d), implying that the displacement of a cell is contributed additively by all the cells proximal to it. Such a patterned displacement should be expected given the growth patterns, and the linear slope implies that all the cells contribute equally to the overall displacement. This orderly cell displacement translated into cellular speed (Supplementary Fig. 3d), with the same correlation pattern relative to cell initial positions (Supplementary Fig. 3c). Importantly, the equal contribution from each cell to the overall displacement means that the relative speed between nearest-neighbour cells should be the same independent of their positions along the PDA (Supplementary Fig. 3e).

To explore the nature of the cooperative behaviour suggested by the trajectory analysis at the individual cell level, we employed a cell-based reference system to analyse the distance change between neighbouring cells. The mean of the distance between a cell and every other cell was calculated at each time point. This yielded unimodal distributions of the mean distances, with similar shapes at all time points, strongly suggesting that cells were displaced away from each other at a global level (Fig. 2e,f). Similar to cell displacement behaviour, cell-cell distance change showed a larger spread along the *y* axis, direction of tissue elongation.

### Conservation of cell spreading behaviour across avian embryos

To determine whether the PZ cell displacement pattern is conserved across avian embryos, the same methodology was applied to analyse metacarpal elongation in transgenic quail embryos constitutively expressing H2B-mCherry in their cell nuclei^19^, (Supplementary Fig. 5a,b; Supplementary Movies 7–9). We found similar cellular trajectories (Supplementary Fig. 5c) and cell-cell distance change (Supplementary Fig. 5f,g), with cellular speed (Supplementary Fig. 3f–h) and displacement lengths (Supplementary Fig. 5d) increasing in an additive and homogeneous manner in the *y* direction. In quail, the two distinct phases of cell displacement occurred with slightly different timing, with cell trajectories that were first displaced distally, then bidirectionally, followed by another phase of distal displacement (Supplementary Fig. 5c). These subtle differences in timing between quail and chick might be the product of species-dependent differences in the timing or patterning of long bone growth, in a largely conserved elongation mechanism.

### CE cannot account for cell spreading

**Figure 3:**
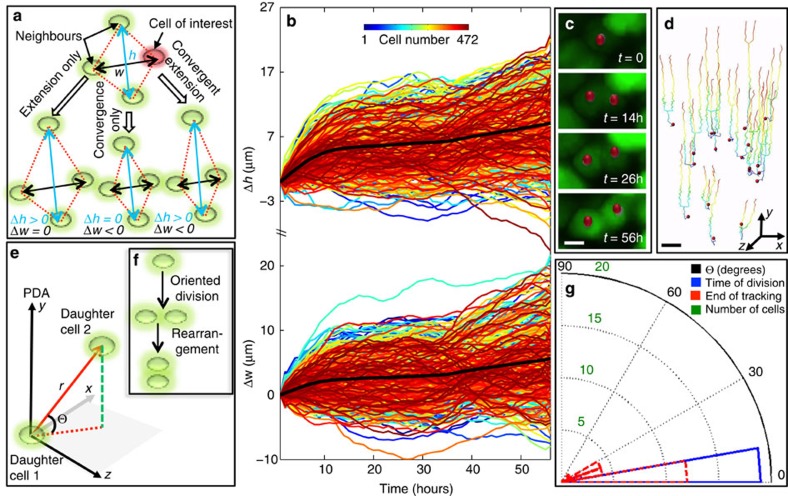
Convergent-extension and mitotic division cannot account for cell spreading. (**a**,**b**) Polygon analysis of convergent-extension (CE). (**a**) Schematic diagrams of polygon analysis. A polygon was drawn for every cell (red) by choosing three nearest neighbours (green). Change in the height (Δ*h*) and the width (Δ*w*) of the polygon depends on the nature of cell motion with respect to its neighbours. (**b**) Δ*h* and Δ*w* of the PZ cells during growth (each coloured line shows the changing value of a single polygon). The increase in the mean of both Δ*h* and Δ*w* (thick black line) over time suggests no significant CE during the observed time window (*n*=472 cells, Supplementary Fig. S4e). (**c**–**g**) Angle analysis of dividing cells. Several time frames of one representative dividing cell expressing GFP (red dots) are presented (**c**), and the trajectories of all dividing cells were mapped (**d**) (*n*=17 cells). To analyse their trajectories, a polar coordinate system was defined (**e**) with *r* and Θ as the distance and angle with respect to *xz* plane between two daughter cells, respectively. If a cell divides along or orthogonal to the PDA, Θ at the time of division is 90 degrees or 0, respectively. If daughter cells undergo rearrangement after orthogonal division (**f**), Θ should undergo significant increase over time. (**g**) Θ was measured for all dividing cells and a polar histogram was employed to show that all Θ were below 15 degrees at the time of division (blue), ruling out the possibility of oriented cell division along the PDA. The fact that all Θ were below 30 degrees by the end of tracking (red) further excluded the possibility of daughter cell rearrangement afterwards (**g**) (*n*=17 cells). Scale bars, (**c**) 10 μm, (**d**) 50 μm.

What, then, are the cellular processes accounting for the observed anisotropic cell spreading and metacarpal elongation? Previous qualitative studies have proposed an important role of CE in controlling cartilage morphology^10^. CE achieves elongation by cell-cell intercalations orthogonal to the growth axis, resulting in the narrowing and lengthening of the embryonic axis of the frog embryo, for example refs 20, 21. As our cell-cell distance analyses indicate extension in all directions (Fig. 2e,f; Supplementary Fig. 5f,g), CE could only be a significant contributor during metacarpal elongation if changes in other cellular morphogenesis compensates for the convergence. To definitively test the contribution of CE, we performed a polygon analysis, in which the relative motions of all cells with respect to their immediate neighbours are tracked over time (Fig. 3a). If CE takes place, the increase in height (along the PDA) of the polygon should accompany the decrease in its width. Using this criterion, our analysis showed that only 10% of the cells underwent CE, while 87% underwent extension-extension, that is, extension in both *x* and *y* directions (Fig. 3b, Supplementary Fig. 4e).

### Mitotic division cannot account for cell spreading

We next tested the ability of mitotic division to explain the growth observed by our quantitative imaging. We identified the cells that underwent mitosis in chick with the cytoplasmic-GFP (Fig. 3c,d; Supplementary Movies 5 and 6) and in quail with the nuclear H2B-mCherry (Supplementary Movies 10 and 11). In both systems, the mitotic rate was below 7%, which is insufficient to explain the large and homogenous cell displacements. Furthermore, measurements of the division angles (Fig. 3e) confirmed that the divisions were oriented orthogonal to the PDA (Fig. 3g; Supplementary Figs 4a,b and 5e), as suggested by previous static analyses^10^,^22^. These findings make it unlikely that mitotic division is a major contributor to the observed cell displacements.

To test this interpretation, we treated E8 cultured chick metacarpals with the cell-cycle blocker mitomycin, which partially inhibited the progression to mitosis (validated by the reduced expression level of the mitosis marker phospho-histone H3 Supplementary Fig. 4d), and found that the treated tissues elongated normally when scored at E9 (Supplementary Fig. 4c). This supports our conclusion that mitotic division is not necessary for tissue elongation during the time window of our experiment. As ultimately the number of cells in a tissue is expected to contribute to its size, further studies are needed to fully elucidate the precise spatio-temporal relationship between cell division and cartilage growth, through extending the analysis over longer time windows.

### Daughter cell rearrangements cannot account for cell spreading

Although cells displayed a division axis orthogonal to the PDA, it remains possible that the division played a role in elongation, if the daughter cells were to subsequently rearrange so that they are oriented along the PDA (Fig. 3f)^9^,^22^,^23^. To address this possibility, we measured the angles and the distances between daughter cells over time (Fig. 3e); only 10% of the dividing cells underwent significant rearrangement (in both chick and quail; Fig. 3g, Supplementary Figs 4a,b and 5e). As such rearrangements were observed in only a small minority of the cells, this mechanism could not be a significant contributor to observed growth and cell displacement.

### ECM expansion and cell volume enlargement account for cell spreading

**Figure 4:**
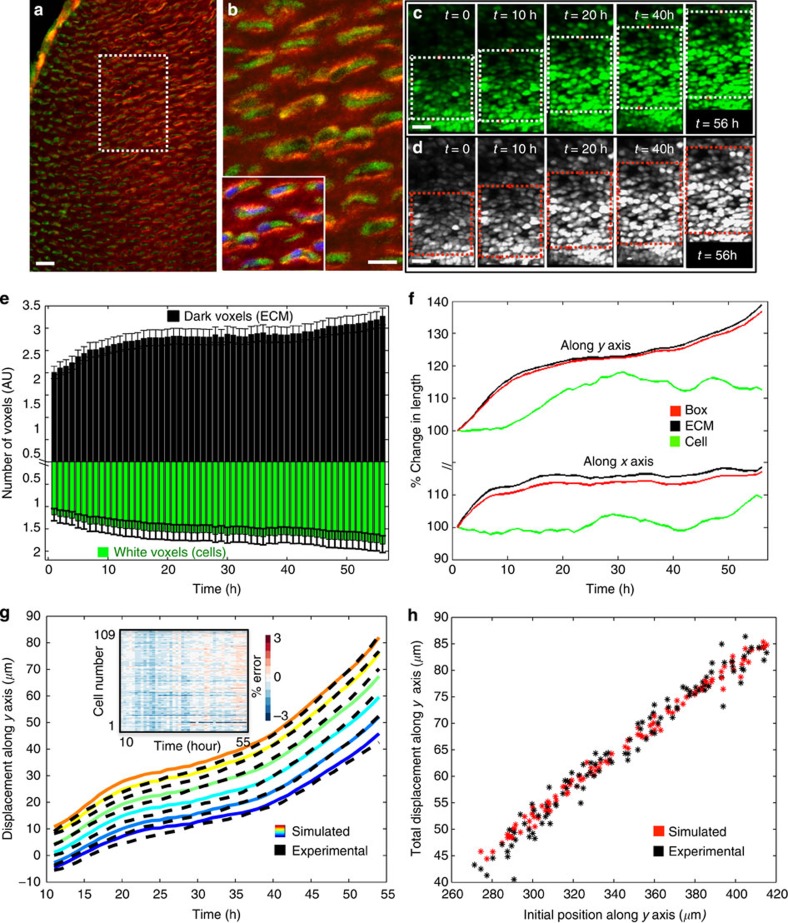
ECM expansion and cell volume enlargement account for cell spreading. (**a**,**b**) *Col2a* antibody staining (red) of frozen sections from the chick metacarpal expressing cytoplasmic-GFP (green). (**a**) Low magnification view. (**b**) Enlarged image of the region in the white box in (**a**). Inset of (**b**) shows sample counterstained with DAPI (blue) to reveal the locations of cell nuclei (three experiments; *n*=4 per experiment). (**c**,**d**) Cell segmentation for voxel analysis. (**c**) Maximum intensity projections of five time points in the 4D live imaging of a chick metacarpal expressing GFP; the region enclosed within the expanding white box selected for voxel analysis, based on the same four cells on the boundaries (red dots). (**d**) Corresponding binary images provided clear identification of voxels as either ECM (black) and cell (white) volume. (**e**,**f**) Voxel analysis. (**e**) Total count of the number of dark and white voxels, denoting the volume occupied by ECM and cells, respectively, shows the expansion of both ECM and cell volume. (**f**) Decomposition of the increase in ECM and cell length along the *x* and *y* axes, expressed as the percentage of the length at *t*=0 (100% denotes no change). (**g**,**h**) Results of computer simulations of cell trajectories, based on the model of tissue growth described in text and depicted schematically in Fig. 5. (**g**) Overlapping simulated and experimental (tracked) cell trajectories along the *y* axis depicted for six randomly chosen cells. Heat map in the insert depicts the errors of all simulated trajectories as a percentage of the experimental values showing that the absolute errors are always below 3%. (**h**) Total cell displacement length (*t*=55 h) along the *y* axis of all simulated cells are plotted against their initial *y* positions, displaying similar distribution pattern to the experimental ones; *n*=109 cells in (**c**–**e**). Scale bars, (**a**,**b**) 15 μm, (**c**,**d**) 50 μm.

Having ruled out major roles for CE, mitotic cell division and daughter cell rearrangement, we addressed the possible roles of cell volume enlargement and intercellular space expansion^11^,^12^,^13^. One of the major components of the ECM filling the intercellular space in the PZ is *col2a* (Fig. 4a,b)^1^,^2^. As 97% of the PZ cells in our imaged chick metacarpals were labelled with cytoplasmic-GFP (Supplementary Fig. 2), the dark area between green cells offers a clean means to recognize the space occupied by ECM, thus allowing us to simultaneously determine the contributions of both cell and ECM volumes. We selected a region in the distal PZ (white box in Fig. 4c) and segmented the image into cell (GFP bright) and ECM (GFP dark) (Fig. 4d); the results showed a 62% increase in ECM volume and a 27% increase in cell volume (Fig. 4e). Decomposing these changes along the *x* and *y* axes revealed that both the ECM and the cells displayed anisotropic expansion, mainly along the *y* axis (Fig. 4f). These changes are consistent with the increase of both height and width in the earlier polygon analysis (Fig. 3a,b). Compared with 12% increase in cell size along the *y* axis (and 10% along the *x* axis), there was a 40% increase in ECM in the same direction (and 20% along *x* axis). Thus, the increase in the total volume of the selected region results from increases in both volumes, with ECM expansion playing the more significant role.

**Figure 5:**
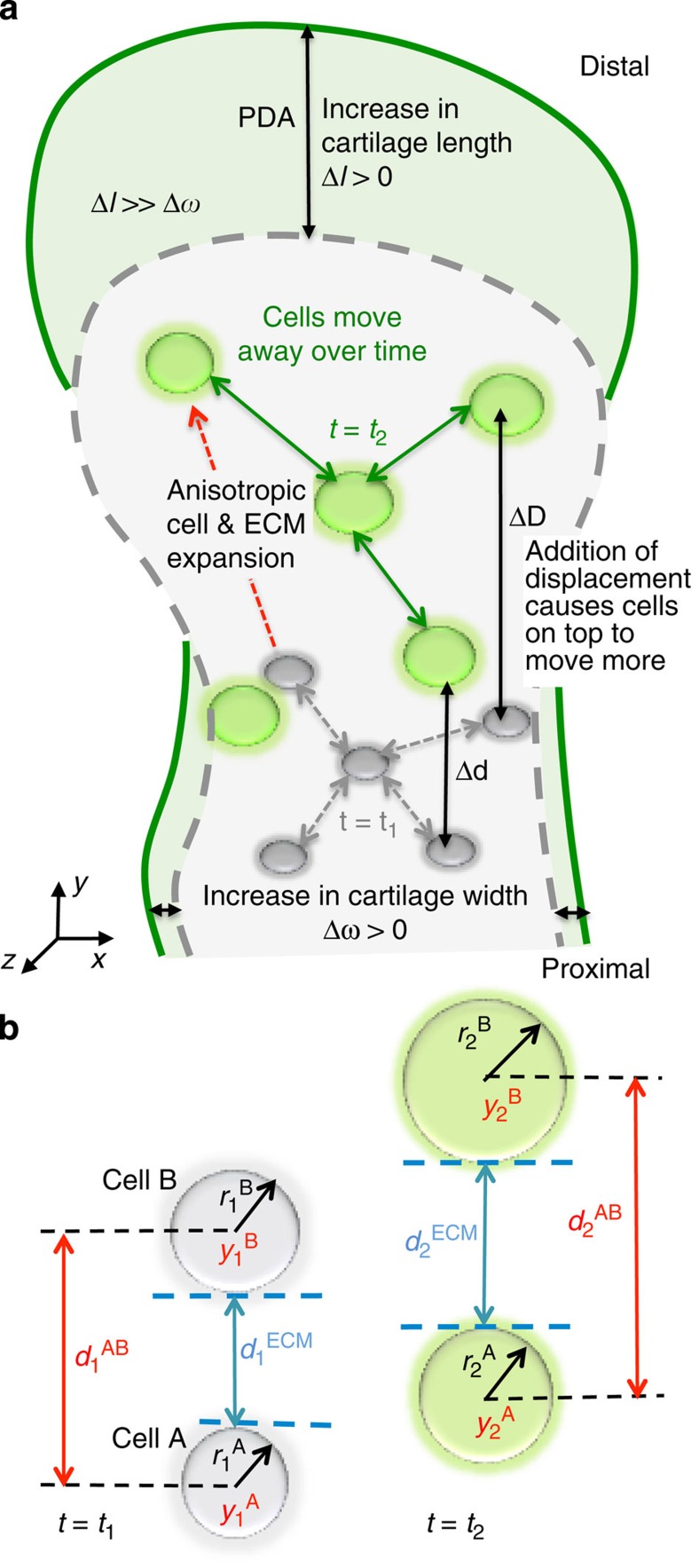
A quantitative model of embryonic cartilage elongation. (**a**) The overall picture of cartilage growth: cell spreading contributed by the anisotropic expansion of ECM and cell volume (cells depicted in grey at *t*_1_ and in green at *t*_2_) results in more increase in length than width of the tissue (from the dashed grey outline at *t*_1_ to the solid green line at *t*_2_). (**b**) Cartilage elongation is a cumulative effect from ECM and cell size increases along the *y* axis, as depicted for a pair of nearest-neighbour cells A and B, at two different time points *t*_1_ and *t*_2_. At time *t*_1_ (*t*_2_), cells A and B are at positions 
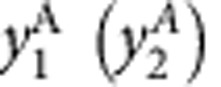
 and 
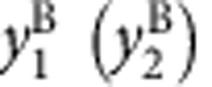
, and have radii 
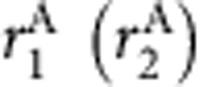
 and 
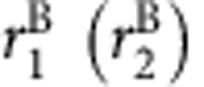
, respectively. The distance between their surfaces, 
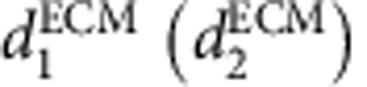
, represents the span of ECM between the two cells. As the cells transit from *t*_1_ to *t*_2_, the increase in centre-centre distance 
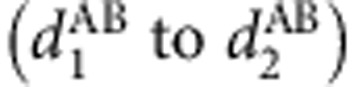
 is the sum of the corresponding increases in ECM 
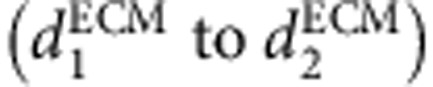
 and the increase in cell sizes 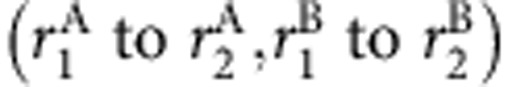.

To test the sufficiency of the observed increases in cellular and ECM volumes to account for the experimental observed cell trajectories, we performed computer simulations based on a model where the cartilage tissue growth and the accompanying cell displacement depends solely on ECM and cellular volume increases (Fig. 5). Using as inputs into the model the measured rates of cell volume and ECM volume increases (the slopes of the graphs in Fig. 4e), and the initial positions of all the cells determined by our cell tracking, we were able to accurately predict the positions of the cells over time (Fig. 4g,h, Methods). The resultant simulated cell trajectories overlapped well with the experimentally observed trajectories within <3% absolute error, for all tracked cells over the experimental time window (inset of Fig. 4g,h), suggesting that the measured cell spreading along the PDA can be sufficiently explained by the expansion of ECM and cell volumes.

## Discussion

Our dynamic analyses offer several novel insights into PZ cell' behaviours during embryonic cartilage elongation: first, the PZ cells show progressive anisotropic spreading mainly along the PDA; second, the relative speed between individual cells are constant and this homogenous behaviour additively contribute to the orderly cell motions observed at a global level; third, mitotic cell divisions, daughter cell rearrangements and motions of CE all take place, but are minor contributors to cellular trajectories; fourth, cell spreading is largely due to the expansion of the ECM, with a secondary contribution from increases in cell volume.

Our data reveal a strong correlation between cell spreading distance and the growth rate of the cartilage. In the tracked PZ cells (Fig. 1g), the distance between the cells at the most distal end and the most proximal end increases by 39% between time zero and the end of the recorded time lapse (The net displacement difference is 85 μm in Fig. 2c, *y* axis, while the length of the region at time zero is 220 μm in Fig. 2d, *x* axis.). During the same period of time, the entire metacarpal extends by 31% (growth of 500 μm from an initial length of 1,600 μm), as quantified by the transmitted-light images taken with lower magnification (Fig. 1e,f). Thus, the percentage change observed in the tracked PZ cells displacement (39% increase) was higher than the entire tissue growth (31% increase); this was expected, as other regions of the metacarpal (for example, the resting zone) might not undergo as much growth as the PZ. Overall, this signifies that the cellular displacement tracked and quantified in our study is relevant to the growth of the entire tissue.

A central task of modern biology is to understand how cells build a tissue in a 4D context^24^,^25^,^26^. Live imaging provides a unique approach to meet this challenge due to its noninvasive ability to collect longitudinal data from cells embodied in their normal context of the live tissues^18^,^19^,^20^. Live imaging generates large data sets, which can be unwieldy to analyse, and biological interpretation of such complex numerical data sets requires quantitative and statistically robust toolsets^27^,^28^,^29^. Here, we have developed a suit of custom software tools to meet this challenge, providing a means for analysing relative distance change between individual cells (Fig. 2e,f), CE (Fig. 3a,b), mitotic division orientation and daughter cell rearrangement (Fig. 3e–g), the rate of ECM deposition and cell volume change (Fig. 4c–h). We showed that cell behaviour during skeleton shaping can be systematically dissected using these quantitative approaches, which can also be adapted for studies of diverse morphogenetic events with minor modifications.

Our trajectory analysis reveals phased cell spreading along the PDA. Cells in the lower PZ displayed proximalwards motion in the first 10 h, and, thereafter, all trajectories coursed distally (Fig. 2c, *y* axis). Interestingly, there is a correlation between the observed cell spreading behaviour and the velocity of ECM deposition. During the first 10 h, the rate of ECM deposition is the highest along both the *x* and *y* axes (as suggested by the slope of the black lines in Fig. 4f). After 10 h, the majority of ECM increase is along the *y* axis (as suggested by the nearly flat plateau of the black line along *x* axis in Fig. 4f). This timing matches well with the orientation and timing of cell spreading (Fig. 2c, *y* axis), indicating that the velocity of ECM deposition is linked to cell displacement behaviour. Determining whether this strong correlation reflects a causal relationship will require a new generation of experiments using our toolset.

Previous work analysing retroviral-based clones of cells with static imaging suggested that the PZ cells undergo a stereotyped rearrangement after division to create elongated ranks of related cells^22^. Here we observed such cell rearrangement at a very low frequency: four mitotically related cells were seen to begin such rearrangements (Fig. 3g). This low frequency of cell rearrangement out of the 472 cells we analysed might be due to the shorter timescale in the current experiments. The virally labelled clones of cells in chick humerus^22^ were analysed 5 days after viral infection. In that study, the typical labelled patch consisted of about four cells, suggesting a slow cell division rate and permitting the subsequent rearrangement to be slow. Our live imaging of the isolated metacarpal lasted for only 55 h. Alternatively, it may be that the formation of ranks of cells takes place earlier in the metacarpals, while cell movement at later stages is restricted due to the different microenvironment. In the future, it will be important to examine the expression of *col2a* and other ECM proteins during different developmental stages.

A recent report of live imaging analysis in a craniofacial cartilage showed that cell rearrangements take place by two daughter cells spreading along their interface^23^. However, the slow elongation rate of the cranial cartilage and the mosaic nature of the GFP-labelled tissue used in that study make it difficult to determine the quantitative contributions of this mode of cell rearrangement to skeleton shaping. Thus, even in systems reporting cell rearrangements in limb skeleton^22^ or in cranial skeleton^23^, it seems unlikely to be the driving force for skeleton elongation.

Immunohistochemistry shows that *col2a* fills the intercellular space in the PZ (Fig. 5a,b), and perturbation study demonstrates that the PZ cells are highly misoriented and the cartilage elements are significantly shorter in the PZ of *col2a*-mutant mice^30^. Thus, based on both our circumstantial and published genetic evidence, we propose that *col2a* deposition, cell morphology and cell spreading coordinate with each other to regulate cartilage elongation. The causal relationship among these three factors remains uncertain. One attractive hypothesis is that the elongated shape of the PZ cells might encourage more *col2a* deposition, which in turn might provide the environment that encourages the elongated cell shape. Indeed, the normal orientation of col2a fibres and the cells' polarized alignment are both disrupted in mutant conditions associated with abnormal cartilage architecture and dimensions^31^,^32^. In addition, the correlation among these three factors might be mediated by a response to chemical signalling. Consistent with this possibility, disrupting the normal activity of noncanonical *Wnt* signalling in both mouse and chick embryos results in misarranged PZ cells^8^,^22^,^33^, as in *col2a*-mutant mice^30^. The resultant shorter and wider cartilage elements further suggest an altered cell spreading behaviour. Future studies, examining the consequences of such alteration in *col2a* and *Wnt* signalling, may help shed light on the relative roles played by physical interactions, the ECM and chemical signalling in the creation of proper tissue architecture and morphology during skeletogenesis.

In this study, 4D imaging and systematic segregation of distinct cellular processes revealed that cartilage elongation in avian embryo is directly controlled by ECM deposition and cell volume enlargement in the PZ. Together with the known dominant roles of these two factors in the HZ in adult cartilage elongation of mammals^11^,^12^,^13^,^34^, our results suggest similar cellular principles underlying skeleton shaping at different developmental stages and in different species.

## Methods

### Viral production and infection

Replication-competent avian retrovirus(A)-GFP was transfected into DF1 cells (ATCC) in 6-cm culture dish using the standard transfection protocol. The transfected cells were split sequentially into 10 and 15 cm dishes. When the cells were confluent in 15-cm dishes, the cell culture medium was harvested once per day for 3 days and was concentrated at 26,000 r.p.m. for 1.5 h. After the spin, the supernatant was discarded while the pellet was dissolved in minimal volume, and further injected into chick (Specific Pathogen-Free chick, Charles River) right forelimbs at E3 (HH 19–20).

### Metacarpal culture and 4D imaging of cartilage elongation

The viral-infected metacarpals were dissected at E8 (HH 32–33) for organ culture. Molten agarose was poured into the fluorodish (World Precision Instruments) and the custom-designed mold was immediately inserted into it. When the agarose was solidified, the mold was pulled out, leaving grooves in the agarose for holding the metacarpals. The metacarpals were submerged in DMEM/F12 growth medium containing 10 mM β-glycerophosphate, 0.2% bovine serum albumin, 50 mM ascorbate acid (Sigma-Aldrich) and 1% glutamine-penicillin-streptomycin (Invitrogen) in a humidified chamber at 37 °C on the stage of the inverted laser scanning microscope (LSM 710, Carl-Zeiss). For imaging chick metacarpal expressing GFP, two-photon laser excitation was used with 26% relative power at wavelength of 900 nm. Optical sectioning was achieved at intervals of 1 μm. For imaging quail metacarpal expressing H2B-mCherry, one-photon laser excitation was used with 26% relative power at wavelength of 561 nm and same optical sectioning.

### Cell segmentation and tracking

The 4D images were imported into IMARIS 7.6.4. The chick metacarpal expressing GFP had a thickness of 193 μm. The middle 90-μm region spanning both stem and loop parts was digitally sliced into three sections along the imaging axis (*z* axis) (Fig. 1h). Three-dimensional spot segmentation of cells and subsequently 4D tracking of cells were performed for each section individually. The cell trajectories from all three sections were combined for quantitative analysis in MATLAB R2013b. Four-dimensional image of quail metacarpal expressing H2B-mCherry had sparse enough signal due to nuclear labels (as opposed to ubiquitous cytoplasmic label in chick). In addition the relatively low penetration of the one-photon excitation only generated images with good resolution for a thickness of 80 μm. Therefore, digital slicing of this data set was not required, and all the nuclei were segmented and tracked using IMARIS 7.6.4.

### Cell displacement length measurement

Displacement lengths were calculated by subtracting the coordinates at time *t*=0 and a given time. The speed was calculated as the distance moved by a cell (difference of coordinates along *y* axis) over time (1-h intervals).

### Analysis of cell-cell distance distributions

On the basis of the coordinates of the tracked cells, center-to-center distance between a cell and every other cell was measured, the distribution of which was used for calculating the mean distance along different orientations. This process was repeated for all the cells and a distribution of average distances was plotted for each time point to depict the shifts in distances over time. The mean of each distribution was further calculated and plotted over time to show statistically average behaviour of all the cells over time.

### Analysis of daughter cell rearrangement

Figure 3e showed the definition of the 3D distance (*r*) and the 3D angle (Θ) between two daughter cells. The axis direction is the same at all time points as defined in Fig. 3d, but the origin is fixed on one of the daughter cells. Changes (increase or decrease) in *r* denote relative displacement of daughter cells (away or towards) over time. Changes in Θ denote rearrangement of the vector joining the center of daughter cells with respect to the *xz* plane. As the divisions occured at different time points for different cells, the time axis was adjusted with time *t*=1 denoting the moment of division.

### Voxel analysis

The images were imported as Tiff files and cropped based on the coordinates of the four cells (in the upper region of PZ) on the boundaries used as the references for drawing the edges of the box for analysis at all times (Fig. 4c). Cropped images were binarized frame-by-frame based on 10 different values of thresholds around the average voxel intensity of that frame. On the basis of the above thresholds, every voxel was assigned an identity as either belonging to cell or to ECM based on the value of one or zero in the binary image, respectively. This process was repeated for all time points and the total numbers of bright and dark voxels were calculated. To decompose the expansion along *x* and *y* axes, the binary images for each frame at a given time point were segmented. The dimensions of all the bounding boxes obtained from every segmented patch at a given time were averaged along the two axes. The change in this denotes the changes in cell dimensions on average. Subtracting this mean length from the dimensions of the cropping box along corresponding axes at every time denotes the changes in ECM span on an average.

### Computer simulation of cellular trajectories

Given that the majority of cartilage growth happens along the PDA, *y* axis (Fig. 2), we assume that measured rates of change of cell volume and ECM volume (the slopes of the graphs in Fig. 4e) come entirely from the changes along the *y* axis. We thus took these measured rates of change and the initial positions of all the cells determined by our imaging and tracking as inputs into a custom-written MATLAB code to simulate the expected cell trajectories. As depicted in Fig. 5b, at every time point the intercellular distance was increased assuming an anisotropic but uniform change along the *y* axis. New cellular positions were predicted at every time point (based on equation (9) below), which were then iteratively used as inputs into the next time step to construct the trajectory over the total duration of imaging. Note that our simulation did not contain any freely adjustable parameters—the only inputs are the measured rates of change in cell and ECM size, and the imaged and tracked cellular positions. The derivation for the simulation based on inputs from the images is outlined below. On the basis of Fig. 5b we know that


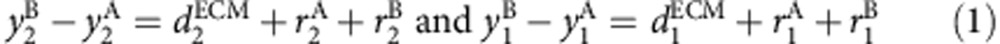


We can write that


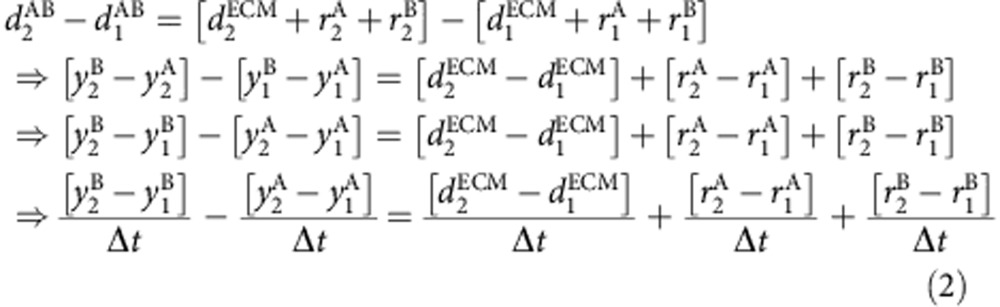



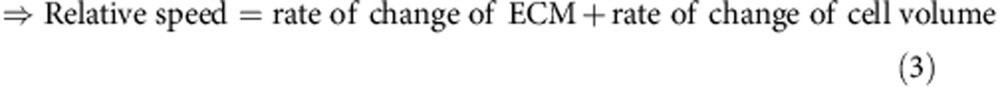


From equation (1), we know that


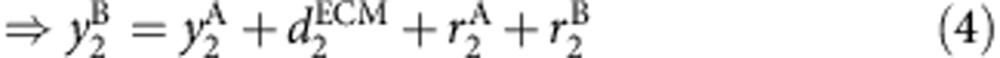



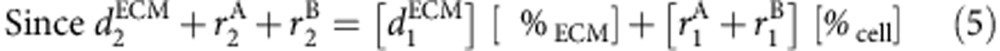



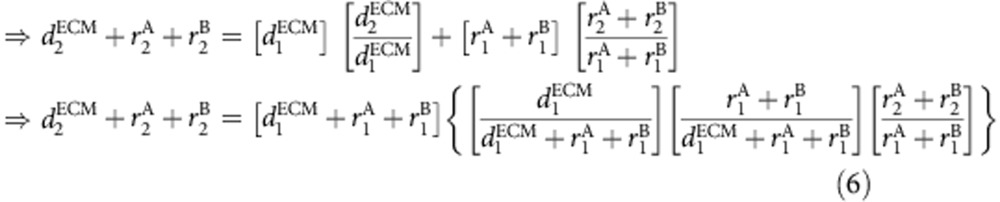



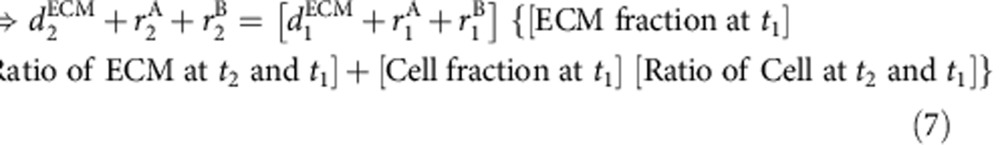


Converting the expression in terms of dark and bright voxels, we get


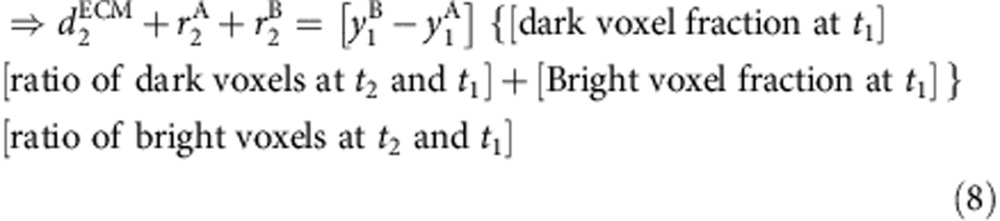


Hence combining equations (4) and (8) we have,


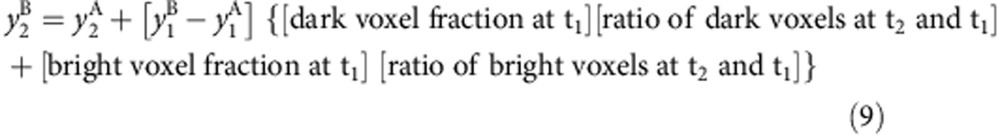


Effectively this also can be simplified to the following


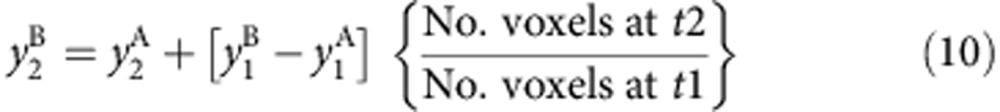


### Isolation of chondrocytes and western analysis

Chondrocytes were isolated from chick metacarpals. After removal of the surrounding perichondrium, the growth plate cartilage was cut into small pieces, homogenized in RIPA buffer and further clarified by centrifugation. The supernatant was used for western blotting with the following antibodies: anti-phosphor-histone-H3 (1:2000; cell signaling), anti-cleaved-caspase (1:2000; cell signaling), anti-β-actin (1:1000, cell signaling) and HRP-conjugated secondary antibodies (1:5000; cell signaling).

### Immunofluorescence and section *in situ* hybridization

Chick metacarpals at E8 were dissected, fixed in 4% paraformaldehyde and frozen in O.C.T. (Tissue-Tek) for making sections (20 μm thickness). For col2a antibody staining, tissue sections were pretreated with 1 mg ml^−1^ pepsin in 0.1 N HCl and blocked in 10% fetal bovine serum before incubating with anti-col2a (1:50, DSHB) and further anti-mouse alexa-488 (1:250, Invitrogen). Phalloidin alexa-546 (1:100, Molecular Probes) was used for counterstaining. For section *in situ* hybridization, tissue sections were hybridized to anti-chick Ihh RNA probes labelled with digoxigenin. Tissue sections of the post-imaging chick metacarpal (expressing GFP) were stained with phalloidin alexa-546 (1:100, Molecular Probes). The number of green (2483 PZ cells) and red signals (2560 PZ cells) of the illuminated half of the tissue was counted using ImageJ.

## Author contributions

All the authors designed the experiments, and Y.L., T.V.T. and V.T. initiated this project. T.V.T designed the mold for organ culture and helped optimize imaging conditions. Y.L., D.S.K. and T.V.T. performed organ culture and live imaging. V.T. and Y.L. performed quantitative analysis. R.L. constructed the transgenic quail. Y.L. performed all other experiments. Y.L, V.T. and S.E.F. wrote the manuscript.

## Additional information

**How to cite this article:** Li, Y. *et al*. Dynamic imaging of the growth plate cartilage reveals multiple contributors to skeletal morphogenesis. *Nat. Commun.* 6:6798 doi: 10.1038/ncomms7798 (2015).

## Supplementary Material

Supplementary Figures Supplementary Figures 1-5

Supplementary Movie 1 Transverse view of chick metacarpal (GFP, green) covering the lower region of the RZ, the whole PZ and the upper region of the PHZ with a thickness of 193μm. The cells on the surface of the tissue are perichondrium cells. Scale bar: 50 μm

Supplementary Movie 2 A transverse view of section 1 (see Fig. 1h) of Supplementary movie 1. Scale bar: 50 μm

Supplementary Movie 3 Live imaging of cellular motion in chick metacarpal (GFP, green) with segmented cells shown in red. Scale bar: 40 μm

Supplementary Movie 4 Trajectories of cellular motion in chick metacarpal (cells segmented in red) with tracks color-coded according to time. Scale bar: 40 μm

Supplementary Movie 5 Live imaging of mitotic cell division in chick metacarpal (GFP, green) with dividing cells segmented in red. Scale bar: 15 μm

Supplementary Movie 6 Trajectories of dividing cells in chick metacarpal (cells segmented in red) with tracks color-coded according to time. Scale bar: 50 μm

Supplementary Movie 7 Transverse view of the quail metacarpal (H2B-mCherry, red) showing nuclei in the lower region of the RZ, the whole PZ and the upper region of the PHZ with a thickness of 80μm. Scale bar: 50 μm

Supplementary Movie 8 Live imaging of nuclei motion in quail metacarpal (H2B-mCherry, red) with segmented nuclei shown in green. Scale bar: 30 μm

Supplementary Movie 9 Trajectories of nuclei motion in quail metacarpal (nuclei segmented in green) with tracks color-coded according to time. Scale bar: 30 μm

Supplementary Movie 10 Live imaging of mitotic cell division in quail metacarpal (H2BmCherry, red) with dividing cells segmented in green. Scale bar: 15 μm

Supplementary Movie 11 Trajectories of dividing cells in quail metacarpal (nuclei segmented in green) with tracks color-coded according to time. Scale bar: 15 μm
