# Supplementary material for: Dynamic imaging of the growth plate cartilage reveals multiple contributors to skeletal morphogenesis
Source: Nat Commun. 2015 Apr 13;6:6798. doi: 10.1038/ncomms7798 (PMC4403347; doi:10.1038/ncomms7798)
Supplement: Supplementary Figures — 1-5 [file ncomms7798-s1.pdf]

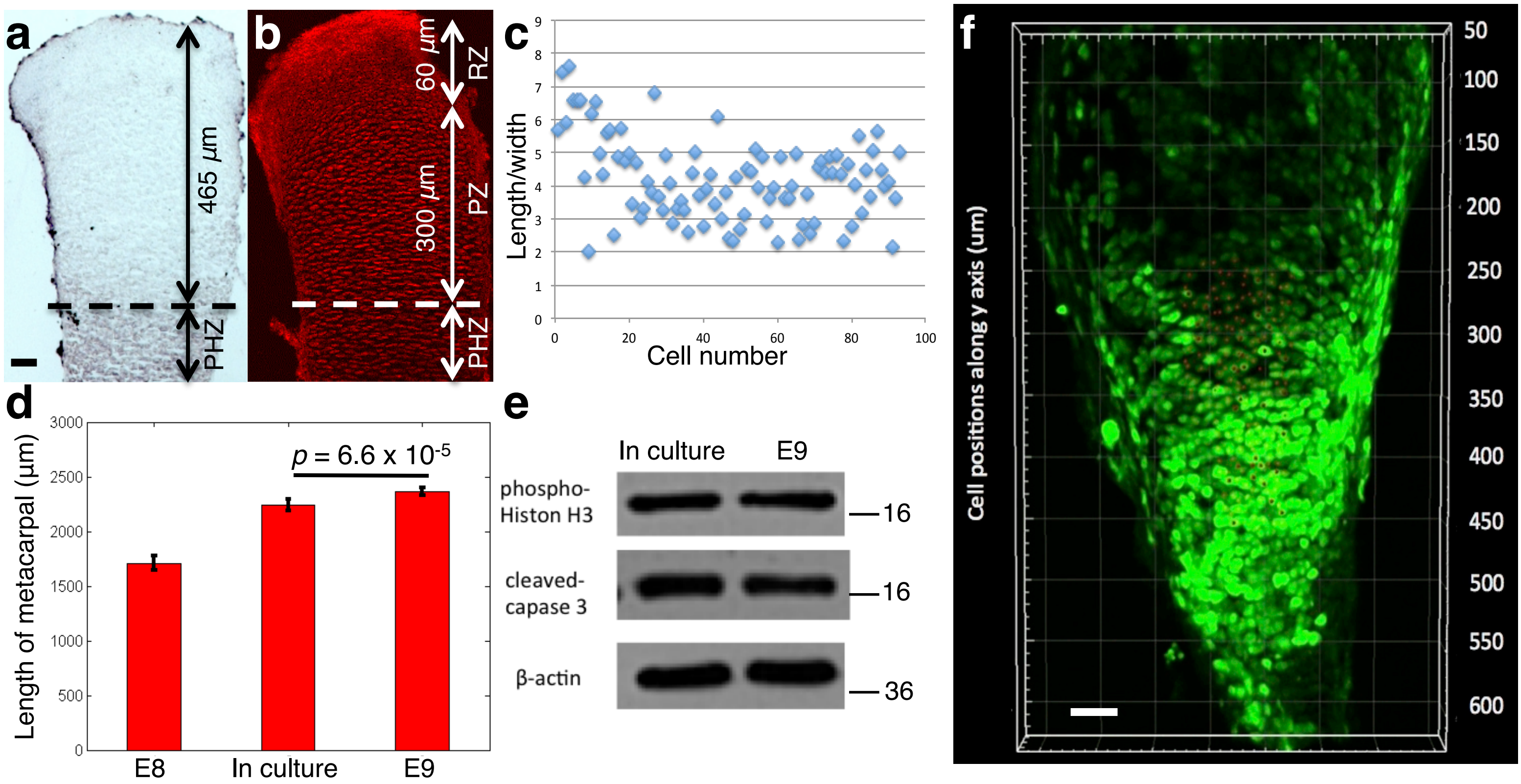

Supplementary Figure 1:
Characterization of chick metacarpal growth from E8 to E9

(a-c) Identification of distinct zones in the sectioned metacarpals. Through section *in situ* hybridization to detect the Indian hedgehog (Ihh) expressing domain, we first identified the border between the PZ and the PHZ **(a)**. The region from the end of the tissue to the border (465µm) comprises of the RZ and the PZ (stdv = 37 µm, n = 14 frozen sections from 3 metacarpals). Since there are no robust molecular markers for the RZ and the PZ cells in chick cartilage, we identified these two populations of cells based on their characteristic morphologies with phalloidin staining on the adjacent sections **(b)**. The PZ cells showed elliptical shape with the average ratio between length and width of 4 (n = 93 cells) **(c)**.

(d, e) Normal chick metacarpal growth in culture.

(d) Chick metacarpals were dissected at E8 and cultured for 24 hours. The cultured tissue and the tissue dissected from E9 embryos showed similar elongation (Student's *t*-test: $p = 6.6 \times 10^{-5}$; 2 experiments, n = 21 metacarpals per experiment).

(e) Western blot analysis of total tissue lysates to detect the expression level of phspho-histon-H3 (17 kD) and cleaved-caspase 3 (17 kD) demonstrated normal cell division and apoptosis, respectively, in the cultured tissues (2 experiments, n = 21 metacarpals per experiment, loading control β-actin (42 kD)).

(f) Identification of the PZ cells (red dots) in the metacarpal for live imaging. Based on the information in **a-c**, the cells in the region between 235µm to 455µm along the y axis at t = 0 were segmented as the PZ cells. Scale bars **(a,b,f)**: 50 µm.

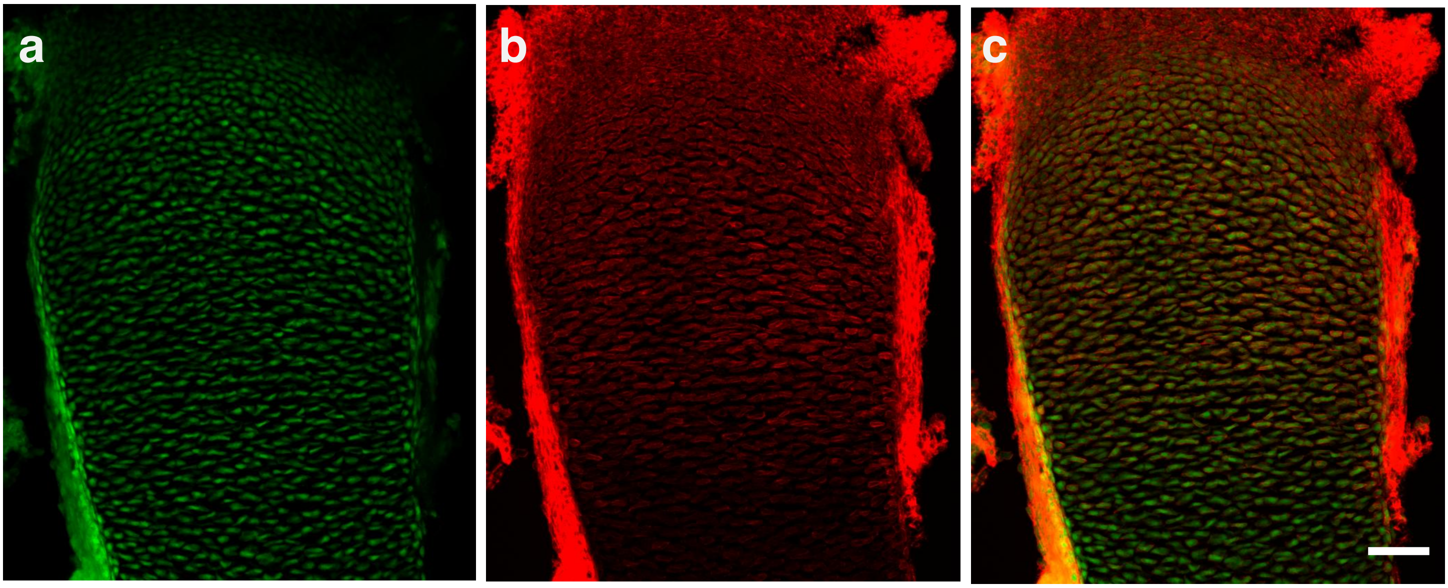

Supplementary Figure 2:
Global infection of the imaged metacarpal by RCAS-GFP
After live imaging, the metacarpal expressing GFP was cryo-sectioned **(a)**, stained with phalloidin **(b)**, and the two images were merged **(c)**. The image presented here is one representative section. (n=8 sections). Scale bars **(a,b,c)**: 50 µm.

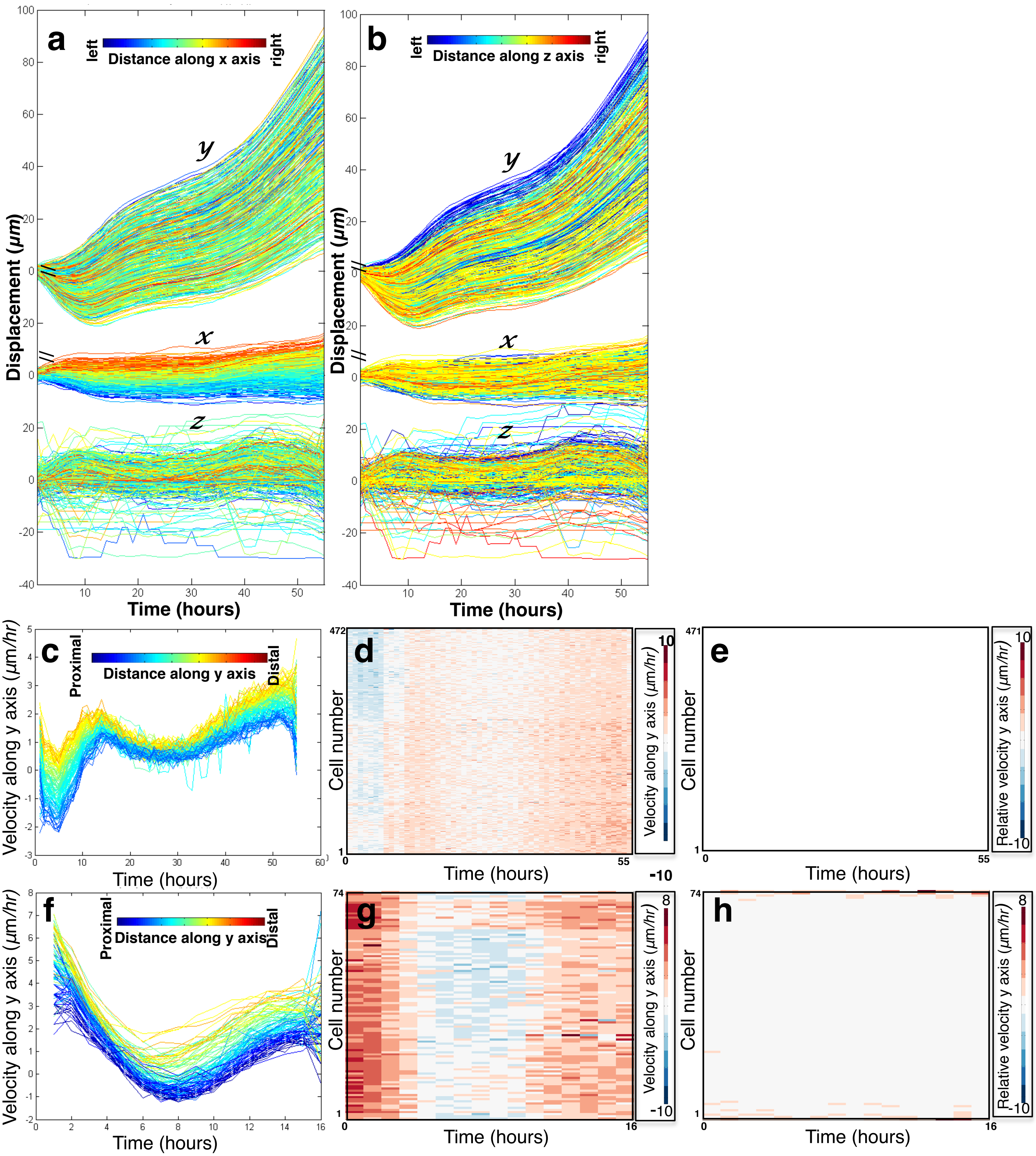

Supplementary Figure 3:
Trajectory analysis of the PZ cell displacement
(a, b) Cell displacement over time relative to cell's initial x **(a)** and z **(b)** positions (color-coded as shown in the top insert) in chick. Note there is no apparent dependence between cell displacements in y direction and their initial x and z positions.**(c, f)** Cellular velocity along the y axis changed accordingly to their initial y positions in chick **(c)** and quail **(f)**. **(d, e, g, h)** Heat map of cellular velocity **(d, g)** and relative velocity **(e, h)** along the y axis in chick **(d, e)** and quail **(g, h)**. The relative velocity was calculated between every cell to the one immediately above it. Note the relative velocity of most cells was close to zero, indicating homogeneous behavior of the entire cell population.

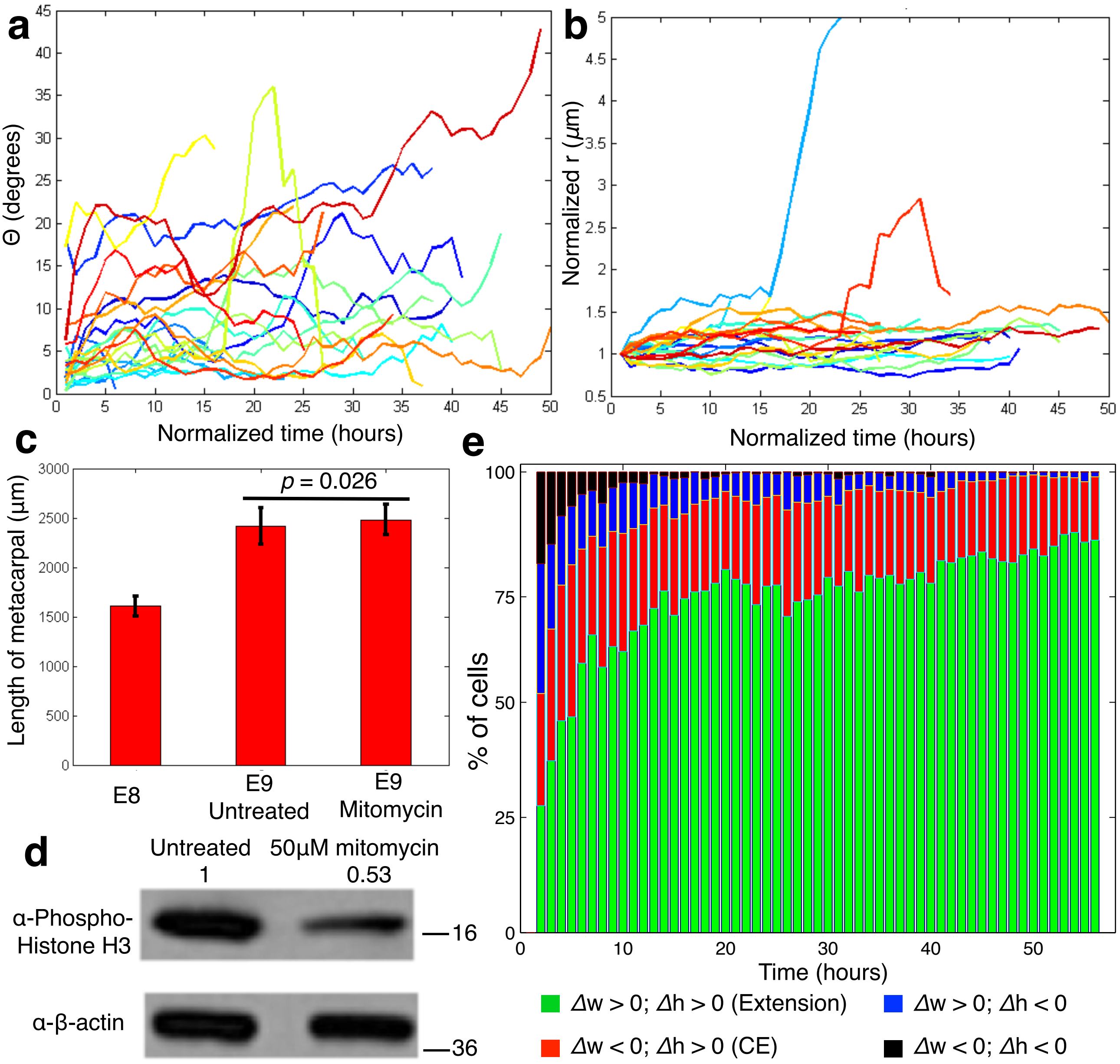

Supplementary Figure 4:
Convergent extension and mitotic division cannot account for cellular trajectories
(a, b) Angle and distance analysis of dividing cells based on the coordinate system in Fig. 3e. The time axis was adjusted with t=1 hour denoting the moment of division. Each color denotes a distinct pair of daughter cells. **(a)** The values of the angle (Θ) between most daughter cells were below 30 degrees over time. **(b)** The normalized distances between most daughter cells (r) (normalized to the respective distances at t = 1 hour) remain near 1 over time indicating their parallel movement during the imaging window. The small values of both Θ and r therefore rule out the possibilities of daughter cell rearrangement.
(c, d) Inhibiting mitotic division did not affect metacarpal elongation. **(c)** Cultured chick metacarpals were treated with 50 µM mitomycin for 24 hours and the average length of the treated and untreated tissues are similar (Student's *t*-test: *p* = 0.026; 2 experiments, n = 16 metacarpals per experiment). **d**, Western blot analysis of total tissue lysates to detect the expression level of phspho-histon-H3 (17 kD) confirmed the inhibition of mitotic division (loading control β-actin (42 kD)).
(e) Majority of cells underwent extension in both x and y directions (green), while only approximately 10% of cells underwent convergent-extension (CE) (red), according to polygon analysis (Fig. 3a). Δw and Δh denote the change in width and height of the polygon formed by four nearest neighbor cells after each time step. Polygons were counted for various combinations of positive and negative values of Δw and Δh and represented in different color bars as shown in the figure.

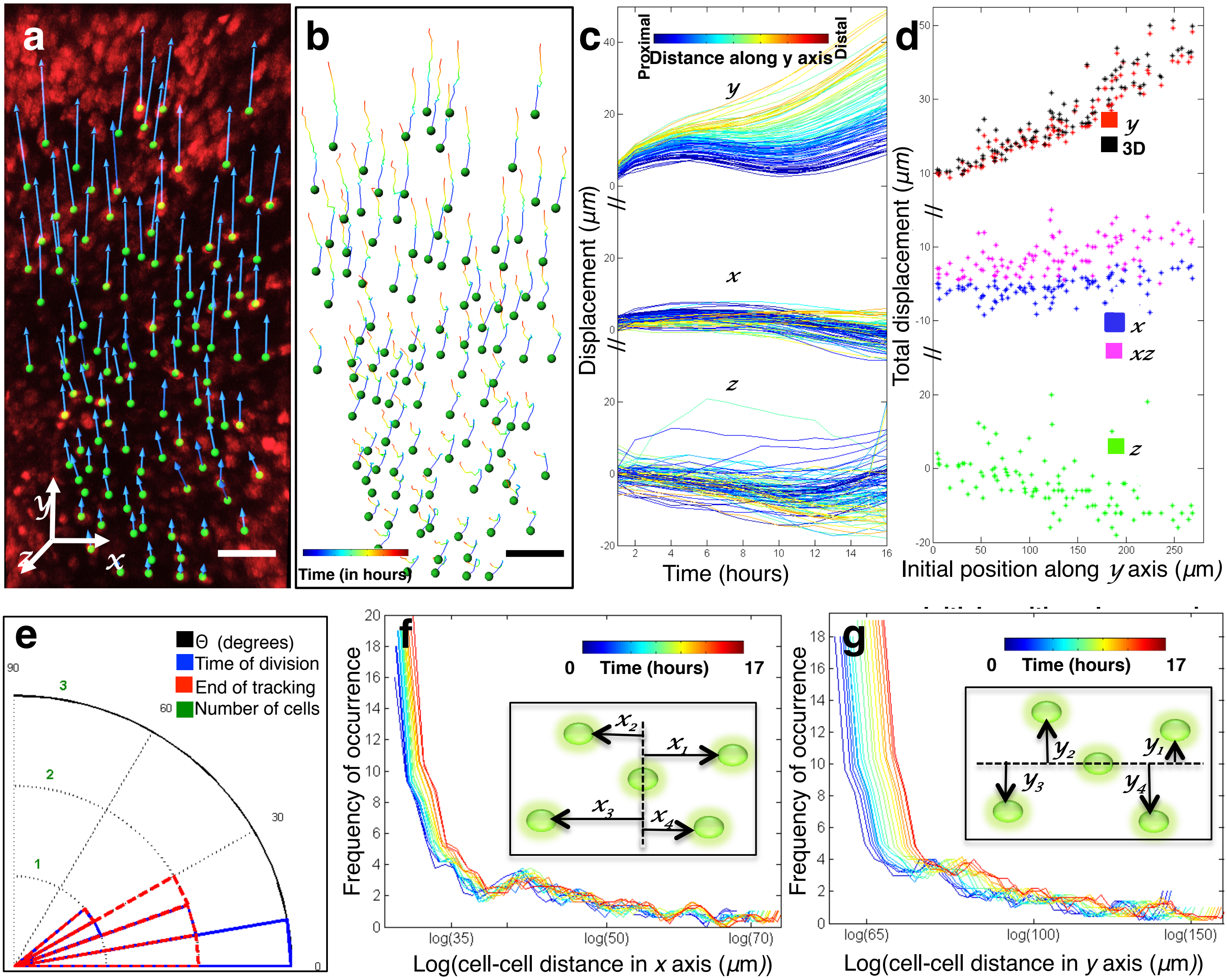

Supplementary Figure 5:
Quail PZ cells exhibit similar spreading displacement and orthogonal division
(a,b) Nuclei in the quail metacarpal expressing H2B-mCherry (red) were segmented (green dots), and their net displacement vectors (blue lines in **a**) and trajectories (colored lines in **b**) were mapped, showing strong orientation of the cell displacement towards the distal end of the tissue similar to observations in chick.
(c,d) Cellular trajectory analysis. **(c)** Cell displacement over time relative to its initial y position (color-coded as shown in the top inset), showing the largest displacement along the y axis and smaller along the x and z axes (n = 74 nuclei). **(d)** Total cell displacement (t = 16 hours) along different axes and planes relative to their initial y positions. Cell displacements along the y axis account for most of the displacements in 3D with similar trend according to their initial y positions as in chick (n = 74 nuclei).
(e) Θ was measured for all dividing nuclei and a polar histogram was employed based on the same coordinate system introduced in Fig 3e. Most Θ were below 15 degrees at the time of division (blue), ruling out the possibility of oriented cell division along the PDA. The fact that majority of Θ were below 30 degrees by the end of tracking (red) further excluded the possibility of daughter cell rearrangement afterwards (n = 4 nuclei).
(f,g) Analysis of cell-cell distance change. The mean of center-to-center distance between all possible pairs of nuclei at any given time was measured (as indicated in the insets), and the distributions of those means for all cells over time (color-coded) were plotted along the x **(f)** and y axes **(g)** on a semi-log scale (to amplify the increase in mean over time graphically). More increase in cell-cell distance along the y axis as compared to the x axis implies an anisotropic spreading behavior of cells (n = 74 nuclei). Scale bar **(a)**: 50 μm.
